# Personal

**Published:** 1906-01

**Authors:** 


### PERSONAL.

Dr. L. S. McMurtry, of Louisville, president of the American Medical Association, and Dr. Charles A. L. Reed, of Cincinnati, a former president of that body, will be the guests of Dr. William Warren Potter, Franklin Street, Buffalo, over Sunday, January 28, 1906, being on their way to attend and participate in the Centennial Anniversary Meeting of the Medical Society of the State of New York, at Albany, January 30, 31, and February 1, of which they have been honorary members for the last fifteen years.

Dr. Joseph Price, of Philadelphia, paid a short visit to Buffalo early in January, on his way home from Toledo and Detroit, where he had been to address medical societies and to operate. Dr. Price's time here was too limited to permit him to call upon a number of his professional friends, which he desired to do.

Mr. A. C. Henderson, for the last eight years a representative of Armour & Company's Laboratory Department in Indiana, Michigan and Northern Ohio, has been called into the home office to succeed Mr. A. C. Tobin, who resigns to take up other work.

Dr. Albert H. Briggs, of Buffalo, was elected president of the Medical Society of the County of Erie, at its Annual Meeting, January 9, 1906. Dr. Briggs is an alumnus of the Medical Department of the University of Buffalo, 1867, and has been engaged in professional practice in this city since that time. He is Surgeon to the 65th Regt. N. G. N. Y., with the brevet rank of lieutenant-colonel having been in service more than twenty-five years. Dr. Briggs is also president of the Association of Military Surgeons of the United States, which will meet in Buffalo in September, 1906.

Dr. Gaylord P. Clark, professor of physiology, has been elected dean of the College of Medicine, Syracuse University, vice Dr. Henry D. Didama, deceased. Dr. Clark has been acting as dean for some years, on account of the ill health of Dr. Didama.

---

Dr. Grover W. Wende, of Buffalo, was elected secretary and treasurer of the American Dermatological Association at its recent annual meeting held in New York, December 28, 1905.

---

Dr. John William Ballantyne, of Edinburgh, Scotland, has been appointed professor of midwifery and diseases of women and children in the University of Edinburgh. Professor Ballantyne is an Honorary Fellow of the American Association of Obstetricians and Gynecologists.

